# Chronic kidney disease and cardiovascular risk: Pathophysiology and interventional approaches – systematic review

**DOI:** 10.1097/MD.0000000000046189

**Published:** 2026-05-12

**Authors:** Muhammad Bari Hassan, Sadia Siddique, Sandeep Kumar Maheshwari, Fnu Neelam, Fnu Neha, Bhawna Lohana, Kiran Lohana, Bismillah Athar Dar, Hina Kumari, Raja Pawan, Payal Bai, Sitara Jabeen, Ali Umair, Ali Karim, Zaghum Abbas, Ugal Kishore, Vikram Kumar, Abida Parveen

**Affiliations:** a Department of Medicine, Ibn e Seena Hospital, Kabul, Afghanistan.

**Keywords:** biomarkers, cardiovascular mortality, cardiovascular risk, Chronic kidney disease, renal dysfunction, risk factors

## Abstract

**Background:**

Chronic kidney disease (CKD) is a major public health concern due to its association with increased cardiovascular risk. Patients with CKD, especially those with moderate to severe renal dysfunction, face a heightened risk of cardiovascular events and mortality. This systematic review evaluates the relationship between CKD and cardiovascular risk, focusing on the pathophysiology, risk factors, and clinical outcomes related to cardiovascular disease (CVD) in CKD populations.

**Methods:**

A comprehensive search of electronic databases was conducted for studies investigating CKD and cardiovascular risk. Seven studies, including cross-sectional, and cohort designs, were included in this review. These studies involved a total of 104,576 participants, with diverse stages of CKD and varying cardiovascular risk profiles. Data on renal dysfunction stages, cardiovascular risk factors, and mortality outcomes were extracted and analyzed.

**Results:**

The studies consistently demonstrated that CKD is associated with an increased risk of cardiovascular events, including coronary heart disease and stroke, compared to individuals without CKD. Even early stages of CKD were linked to a higher likelihood of CVD. Participants with CKD exhibited a greater burden of traditional cardiovascular risk factors, including hypertension, diabetes, dyslipidemia, and obesity. The overall mortality rate from cardiovascular causes was also higher in CKD patients, with some studies reporting a two-fold or greater increase in risk.

**Conclusion:**

CKD is strongly linked to an increased cardiovascular risk, with higher stages of renal dysfunction associated with a more significant risk of cardiovascular morbidity and mortality. This review emphasizes the need for early detection and comprehensive management of cardiovascular risk factors in CKD patients. Future research should focus on identifying novel biomarkers and evaluating the long-term efficacy of emerging therapies in reducing cardiovascular risk in CKD populations.

## 1. Introduction

Chronic kidney disease (CKD) represents a significant global health issue, affecting approximately 10% of the population and leading to severe complications, including cardiovascular disease (CVD).^[1]^ As kidney function deteriorates, patients face an exponential increase in the risk of cardiovascular morbidity and mortality, making CVD the leading cause of death in CKD patients.^[2]^ This heightened risk is evident even in the earliest stages of CKD and persists across the disease continuum.^[3]^ Understanding the intricate link between CKD and cardiovascular risk is critical to improving outcomes in this vulnerable population.^[4]^ The pathophysiology connecting CKD and CVD involves both traditional and nontraditional risk factors. Traditional factors such as hypertension, diabetes mellitus, and dyslipidemia are highly prevalent in CKD patients. However, nontraditional factors unique to CKD, including uremic toxins, chronic inflammation, oxidative stress, endothelial dysfunction, and vascular calcification, significantly amplify cardiovascular risk.^[5]^ Furthermore, disturbances in mineral metabolism, such as elevated phosphate levels and secondary hyperparathyroidism, exacerbate vascular damage and contribute to adverse cardiovascular outcomes.^[6]^

Globally, over 850 million people are affected by CKD, acute kidney injury, or are on renal replacement therapy – a figure twice that of diabetes and over 20 times higher than HIV/AIDS – highlighting kidney disease as one of the most prevalent global health issues.^[7,8]^

Despite advances in understanding these mechanisms, managing cardiovascular risk in CKD remains challenging. Standard interventions, including antihypertensive therapy, lipid-lowering agents, and glycemic control, are often less effective due to the complexities of CKD-specific pathophysiology.^[8]^ Moreover, achieving optimal treatment targets is frequently hindered by the presence of comorbidities and the progressive nature of CKD.^[9]^ This systematic review aims to provide an in-depth analysis of the pathophysiological links between CKD and cardiovascular risk while evaluating current and emerging interventional strategies. By synthesizing existing evidence, this review seeks to identify gaps in knowledge, highlight innovative therapeutic approaches, and guide future research efforts. Addressing cardiovascular risk in CKD patients is crucial to reducing mortality and improving their overall quality of life.

## 2. Methods

### 2.1. Study design and search strategy

**Figure 1.**
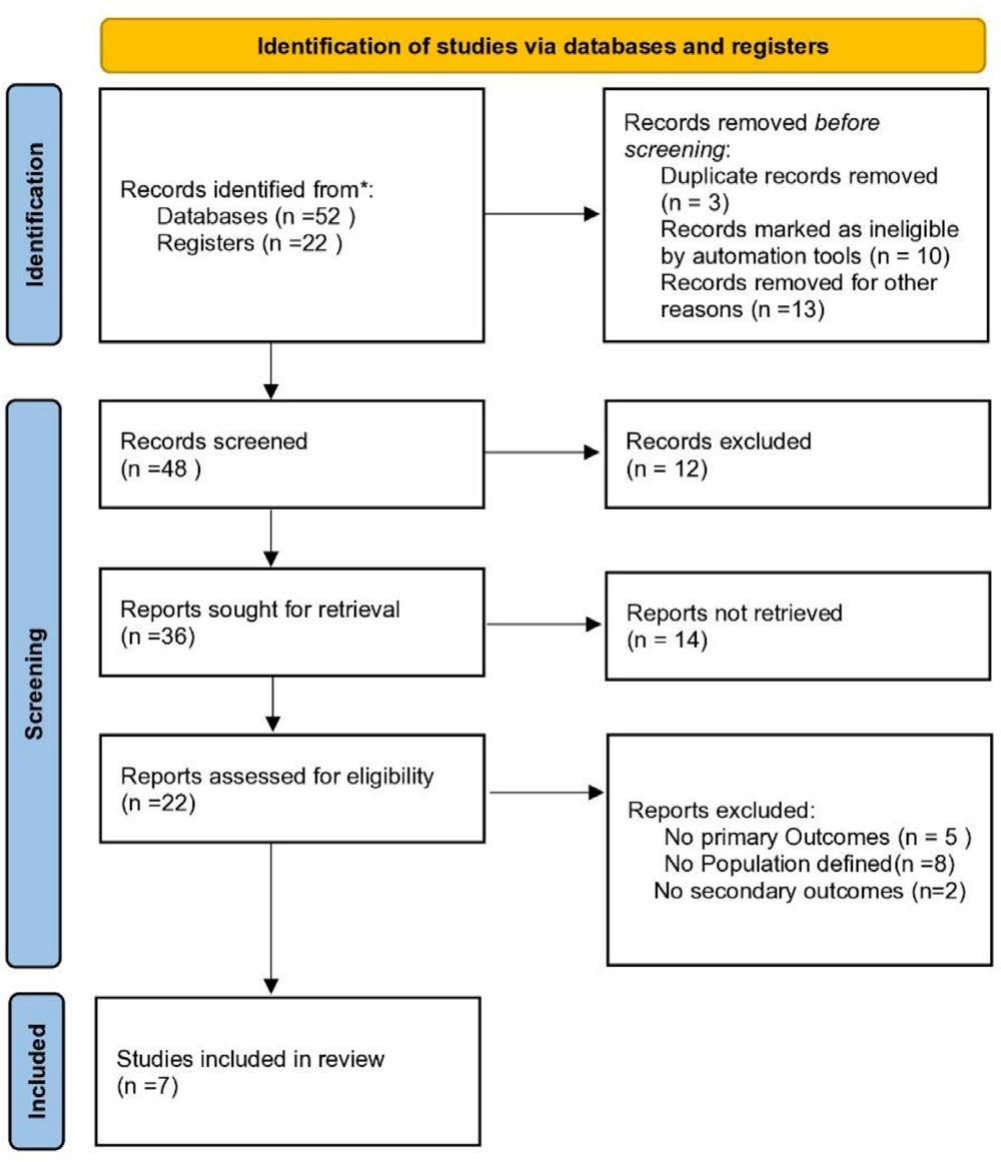
PRISMA flowchart. PRISMA = preferred reporting items for systematic reviews and meta-analyses.

This systematic review was conducted in accordance with PRISMA (Preferred Reporting Items for Systematic Reviews and Meta-Analyses) guidelines (Fig. 1). A comprehensive literature search was performed in major databases, including PubMed, Embase, and the Cochrane Library, to identify relevant studies published up to December 2024. The search strategy used combinations of keywords and MeSH terms such as “CKD,” “cardiovascular risk,” “pathophysiology,” and “interventional approaches,” combined with Boolean operators to refine the results. Additional studies were identified by screening the reference lists of included articles. For transparency and reproducibility, the full search strategy, including detailed search terms and Boolean combinations, is provided as a Table S1 (Supplemental digital content, https://links.lww.com/MD/Q806).

### 2.2. Eligibility criteria

Studies were included if they focused on the relationship between CKD and cardiovascular risk, discussed underlying pathophysiological mechanisms, or evaluated interventional approaches to mitigate cardiovascular complications in CKD. Randomized controlled trials, cohort studies, case-control studies, and systematic reviews were included. Articles were excluded if they were not in English, lacked original data, or focused on non-human subjects.

### 2.3. Data extraction and quality assessment

Data were extracted independently by 2 reviewers using a predesigned extraction sheet. Key information collected included study design, population characteristics, CKD stage, cardiovascular outcomes, and interventional strategies. The quality of included studies was assessed using appropriate tools, such as the Cochrane Risk of Bias Tool for randomized trials and the Newcastle–Ottawa Scale for observational studies. Discrepancies were resolved through discussion or consultation with a third reviewer. The traffic light plot for the risk of bias assessment, showing each study across the 3 Newcastle–Ottawa Scale domains (selection, comparability, outcome/exposure) is shown in Figure S1 (Supplemental digital content, https://links.lww.com/MD/Q806).

### 2.4. Data synthesis and analysis

Findings were synthesized narratively and, where possible, meta-analyzed to quantify the association between CKD and cardiovascular risk or evaluate the effectiveness of interventions. Subgroup analyses were performed based on CKD stage, intervention type, and study design. Publication bias was assessed using funnel plots and statistical tests.

### 2.5. Ethics

No ethical approval was required as only published studies were analyzed. Ethical compliance was assumed. This review followed established PRISMA (Preferred Reporting Items for Systematic reviews and Meta Analyses) 2020 guidelines (Fig. 1), ensuring a rigorous and transparent methodology throughout the process. And no informed consent was required because no new data generated.

## 3. Result

This systematic review includes a total of 7 studies involving a combined total of 107,434 participants. The studies varied in design, including prospective, cross-sectional, and cohort studies, and encompassed a range of CKD stages and cardiovascular risk profiles. The key findings from these studies, show a consistent increase in cardiovascular risk among individuals with CKD, especially as renal dysfunction progresses. The Table 1 summarizes essential details such as the study design, number of participants, follow-up periods, degree of renal dysfunction, estimated glomerular filtration rate (eGFR), cardiovascular risk, and mortality rates for each study. These studies demonstrate that CKD significantly exacerbates cardiovascular morbidity and mortality, with an increased burden of traditional cardiovascular risk factors, such as hypertension, diabetes, dyslipidemia, and obesity, across the CKD population. The data further emphasizes the critical need for early detection and management of cardiovascular risk in CKD patients to reduce associated mortality rates.

**Table 1:** Summary of studies investigating chronic kidney disease and cardiovascular risk.

Author/year	Study design	No of participants	Men/women	Follow -up (yr)	Degree of renal dysfunction	Renal dysfunction (stage)	Estimated glomerular filtration rate (eGFR)	Cardiovascular risk	Cardiovascular risk factors	Mortality rate (any cause)	Quality assessment
Venkatesh L Murthy et al^[10]^ (2012)	Prospective study	866	Men, 435 (50.2)	1.28	Moderate to severe renal dysfunction	CKD stage 3 = 559 (64.6) CKD stage 4 = 106 (12.2) CKD stage 5 = 201 (23.2) Dialysis = 153 (17.7)	≤60 mL/min/1.73 m^2^	2.1-fold increase in the risk of cardiac death	BMI (kg/m^2^) = 28.2 [24.4–33.0] BMI ≥ 30 kg/m^2^ = 347 (40.1) Hypertension = 790 (91.2) Dyslipidemia = 606 (70.0) Diabetes = 388 (44.8) Family history of CAD = 189 (21.8) Tobacco use = 75 (8.7)	155 (17.9%)	**
Bogdan Ene-Iordache et al^[11]^ (2016)	Cross-sectional study	75,058	Men, 46,664 (62%) Women, 28,394 (38%)		559/8155 (7%)		88.4 (21.4) mL/min/1.73 m^2^	General cohort (n = 67,009): 75% low, 16% medium, 10% high risk. High-risk cohort (n = 4250): 49% low, 18% medium, 33% high risk	Hypertension 9153/15,461 (59%), Diabetes 2745/3868 (71%)		***
Marian Goicoechea et al^[12]^ (2005)	Cross-sectional study	128	78 M, 50 F	22.3 mo	Moderate CKD		34.8 ± 13.6 mL/min	10-yr CHD risk % = 15.5 ± 8.2	Diabetes = 41 participants (with + without CV events)	4 died	******
Michael G Shlipak et al^[13]^ (2005)	Prospective cohort study	5808	More were men	8.6 yr	1249 (22%) participants have chronic kidney disease	Stage 1: ≥90 mL/min/1.73 m^2^ Stage 2: 60–89 mL/min/1.73 m^2^ Stage 3a: 45–59 mL/min/1.73 m^2^ Stage 3b: 30–44 mL/min/1.73 m^2^ Stage 4: 15–29 mL/min/1.73 m^2^ Stage 5: <15 mL/min/1.73 m^2^	<60 mL/min per 1.73 m^2^	Traditional risk factors greatly increase cardiovascular death risk in CKD patients	With CKD, LVH: +25/1000; smoking: +20/1000 person-years. Without CKD, Average risk: 16/1000; LVH: +17/1000; diabetes: +15/1000; smoking: +13/1000	32 vs 16 per 1000 person-years (CKD vs non-CKD).	**
Emanuele Di Angelantonio et al^[14]^ (2010)	Prospective population based cohort study	16,958	9134 male and 9769 female participants	24 yr	1210 (7%) of participants had chronic kidney disease		75–89 mL/min/1.73 m^2^	Excess risk of subsequent coronary heart disease even in the earliest stage	Body mass index (kg/m^2^) 25.4 (3.9). systolic blood pressure (mm Hg) 138 (22). History of diabetes 400 (2.4). Current cigarette smokers 8013 (47.3). total cholesterol (mmol/L) 6.48 (1.16)	559 deaths from stroke, 662 deaths from other vascular causes, and 3875 deaths from non-vascular causes were recorded	***
Hiromasa Kitamura et al^[15]^ (2023)	Cross-sectional study	3407	Female sex, %1948 (44.0)				40.3 (23.7–57.7) mL/min/1.73 m^2^	Increased cardiovascular risk factor burden with greater severity of CKD	BMI, kg/m^2^ 22.9 (20.6–25.6). Diabetic nephropathy 364 (10.7), Systolic blood pressure, mm Hg 130 (120–142). History of CVD, %880 (25.8)		***
Nisha I Parikh et al^[16]^ (2006)	Community-based prospective cohort study	5209	Female, 1612 (54%)			96.1% had stage 3 CKD (n = 270), remaining 3.9% of participants, stage 4 (n = 8) stage 5 (n = 3)	Participants with CKD, 18 ± 17 mL/min/1.73 m^2^	The 10-yr CVD risk (≥10%) was 4 times higher in people with CKD than without	Participants with CKD, Systolic blood pressure, 128 ± 18 mm Hg BMI, 28.1 ± 5.4 kg/m^2^ Smoker, 402 (13.5%) Total cholesterol level ≥ 240 mg/dL, 424 (14.2%)		****

The asterisk symbols indicate the quality of the studies included; the greater the number of asterisks, the higher the quality.

BMI = body mass index, CAD = coronary artery disease, CHD = coronary heart disease, CKD = chronic kidney disease, CVD = cardiovascular disease, LVH = left ventricluar hypertrophy.

Venkatesh L. Murthy et al (2012), in a prospective study of 866 patients, demonstrated that those with coronary flow reserve < 1.5 had more than a two-fold higher risk of cardiac death compared to those with preserved flow reserve (adjusted HR 2.1, 95% CI: 1.3–3.5).^[10]^ Bogdan Ene-Iordache et al (2016), in a large cross-sectional study of 75,058 participants, reported that CKD was consistently associated with higher adjusted prevalence of cardiovascular risk factors such as hypertension and diabetes, although odds ratios with confidence intervals were not provided.^[11]^ Marian Goicoechea et al (2005), in a cross-sectional study of 128 patients, identified traditional risk factors including hypertension and dyslipidemia as strong predictors of cardiovascular events in CKD, but did not report adjusted hazard ratios.^[12]^ Michael G. Shlipak et al (2005), using a prospective cohort of 5808 participants, found that CKD conferred an increased risk of cardiovascular mortality independent of traditional risk factors, with adjusted HRs ranging from 1.4 to 1.9 across CKD categories.^[13]^ Similarly, Emanuele Di Angelantonio et al (2010), in a population-based cohort of 16,958 individuals, reported that even mild reductions in eGFR were associated with a higher incidence of coronary heart disease and cardiovascular mortality, with adjusted relative risks of approximately 1.5 (95% CI: 1.2–1.9).^[14]^ Hiromasa Kitamura et al (2023), in a cross-sectional study of 3407 patients, demonstrated that greater CKD severity was associated with an incremental burden of uncontrolled cardiovascular risk factors, though adjusted estimates with confidence intervals were not presented.^[15]^ Finally, Nisha I. Parikh et al (2006), in a community-based prospective cohort of 5209 participants, showed that individuals with CKD had substantially higher 10-year cardiovascular risk compared to those without CKD, with adjusted HRs exceeding 1.5 after accounting for conventional risk factors.^[16]^

## 4. Discussion

CKD is a significant global health issue due to its strong association with CVD, the leading cause of death in this population.^[17]^ The relationship between CKD and CVD is driven by complex pathophysiological mechanisms involving both traditional and nontraditional risk factors. Understanding these mechanisms is essential for improving clinical outcomes through targeted interventions and holistic patient care.^[18]^

### 4.1. Pathophysiological link between CKD and cardiovascular risk

#### 4.1.1. Traditional risk factors

Traditional risk factors such as hypertension, diabetes, and dyslipidemia are prevalent in CKD and play a significant role in cardiovascular risk. Hypertension is not only a major cause of CKD but also exacerbates renal damage through increased pressure on glomeruli and systemic vascular stress.^[19]^ Diabetes accelerates vascular damage through glycation end products and promotes atherosclerosis.^[20]^ Dyslipidemia in CKD is unique, characterized by low HDL cholesterol and elevated triglycerides, which increase the likelihood of cardiovascular complications.^[21]^

#### 4.1.2. Non-traditional risk factors

In addition to traditional risks, CKD-specific factors significantly amplify cardiovascular risk. Chronic inflammation, driven by uremic toxins and oxidative stress, accelerates vascular damage and endothelial dysfunction. Uremic toxins such as indoxyl sulfate and p-cresyl sulfate directly contribute to vascular calcification and atherosclerosis.^[22]^ Furthermore, disturbances in mineral metabolism, including hyperphosphatemia and secondary hyperparathyroidism, result in arterial stiffness and calcification, hallmark features of CKD-associated CVD.^[23]^ Anemia, another common CKD complication due to erythropoietin deficiency, exacerbates myocardial ischemia and heart failure, contributing to poor cardiovascular outcomes.^[24]^

### 4.2. Cardiovascular complications in CKD

#### 4.2.1. Coronary artery disease

CKD accelerates the development of CAD through mechanisms such as endothelial dysfunction, atherosclerosis, and vascular calcification. Patients with CKD often exhibit extensive coronary calcification, leading to a unique presentation of CAD that is less amenable to standard therapies.^[25]^

#### 4.2.2. Heart failure

Heart failure, particularly heart failure with preserved ejection fraction, is prevalent in CKD due to volume overload, left ventricular hypertrophy, and myocardial fibrosis. The bidirectional relationship between CKD and heart failure complicates management, as each condition exacerbates the other.^[26]^

#### 4.2.3. Arrhythmias and sudden cardiac death

CKD patients face an increased risk of arrhythmias, including atrial fibrillation and ventricular arrhythmias, due to factors such as electrolyte imbalances, left ventricluar hypertrophy, and myocardial fibrosis. Sudden cardiac death is a major cause of mortality in CKD and is linked to these arrhythmogenic factors.^[27]^

### 4.3. Interventional approaches

#### 4.3.1. Blood pressure management

Controlling hypertension is critical for reducing cardiovascular risk in CKD. Renin-angiotensin-aldosterone system inhibitors, including angiotensin converting enzyme inhibitors and angiotensin receptor blockers, are first-line therapies due to their benefits in reducing proteinuria and slowing CKD progression.^[28]^ However, treatment resistance is common in CKD, requiring combination therapies and personalized approaches.

#### 4.3.2. Lipid management

Statins are effective in reducing cardiovascular events in early CKD stages, but their benefits in advanced CKD or dialysis patients are limited. Ezetimibe and PCSK9 inhibitors may provide additional benefits, though their use in CKD-specific populations requires further study.^[29]^

#### 4.3.3. Glycemic control

Effective glycemic control in CKD patients with diabetes is crucial for reducing cardiovascular events. Recent advancements, including sodium-glucose cotransporter-2 (SGLT2) inhibitors and glucagon-like peptide-1 (GLP-1) receptor agonists, have shown significant benefits in improving both cardiovascular and renal outcomes.^[30]^ These therapies represent a shift in diabetes management, particularly for CKD patients.

#### 4.3.4. Mineral and bone disorder management

Addressing disturbances in mineral metabolism, such as hyperphosphatemia and secondary hyperparathyroidism, is essential for reducing vascular calcification and cardiovascular risk.^[31]^ Phosphate binders, calcimimetics, and vitamin D analogs are commonly used, but their long-term cardiovascular benefits remain unclear.

#### 4.3.5. Lifestyle interventions

Lifestyle modifications, including dietary changes, smoking cessation, and increased physical activity, are foundational in managing cardiovascular risk. Sodium restriction and adherence to heart-healthy diets like DASH or the Mediterranean diet have demonstrated benefits in CKD patients.^[32]^

#### 4.3.6. Emerging therapies

Novel therapeutic approaches targeting CKD-specific mechanisms are under investigation. Anti-inflammatory agents, such as interleukin-1 inhibitors, aim to reduce chronic inflammation and improve cardiovascular outcomes.^[33]^ Therapies targeting uremic toxins and vascular calcification, including selective phosphate transport inhibitors, offer potential benefits for CKD patients.^[34]^

### 4.4. Challenges in cardiovascular risk management

Managing cardiovascular risk in CKD patients presents unique challenges. Traditional therapies often have reduced efficacy in advanced CKD, necessitating the development of tailored interventions. Additionally, CKD patients are underrepresented in cardiovascular clinical trials, limiting the generalizability of evidence-based guidelines. Multimodal approaches that address the interplay between CKD and CVD are critical for improving outcomes.^[35]^

### 4.5. Future directions

Future directions in managing cardiovascular risk in CKD focus on improving early detection and personalized treatment strategies. Identifying novel biomarkers related to kidney function, inflammation, and vascular calcification will enable better risk stratification and targeted interventions. Additionally, evaluating the long-term benefits of emerging therapies, such as SGLT2 inhibitors and GLP-1 receptor agonists, in CKD populations is essential. CKD-specific clinical trials are needed to develop evidence-based guidelines, as current cardiovascular trials often exclude CKD patients or fail to address their unique needs.^[36]^ A multimodal approach combining pharmacological treatment with lifestyle changes, such as dietary adjustments and smoking cessation, along with personalized medicine tailored to individual patient characteristics, will be critical to optimizing outcomes and reducing cardiovascular burden in CKD patients.^[37]^

### 4.6. Strengths and limitations

This systematic review has several strengths, including a comprehensive and methodologically rigorous approach that adheres to PRISMA guidelines, incorporation of diverse study designs with a large cumulative sample size of over 107,000 participants, and the use of validated quality assessment tools. It offers an in-depth analysis of both traditional and CKD-specific cardiovascular risk factors while highlighting current and emerging interventional strategies. However, certain limitations must be acknowledged. The heterogeneity in study populations, CKD staging criteria, and outcome measures may affect the comparability of results. Most included studies were observational, which limits causal inferences. Additionally, the exclusion of non-English publications introduces potential language bias, and some studies lacked detailed intervention data or CKD stage stratification, limiting the ability to draw nuanced conclusions. Publication bias may also be present due to the reliance on published literature.

## 5. Conclusion

CKD is intricately linked to increased cardiovascular risk through a combination of traditional and CKD-specific factors. Addressing this risk requires a comprehensive understanding of the underlying pathophysiology and the implementation of targeted interventions.^[38,39]^ While advancements in therapy have improved outcomes, significant gaps remain, particularly in advanced CKD.^[40]^ Continued research and innovation are essential to reducing cardiovascular morbidity and mortality in CKD patients, ultimately enhancing their quality of life.^[41]^

## Author contributions

**Conceptualization**: Muhammad Bari Hassan, Sadia Siddique, Sandeep Kumar Maheshwari, Fnu Neelam, Bhawna Lohana, Bismillah Athar Dar, Raja Pawan, Payal Bai, Ali Umair, Ali Karim, Zaghum Abbas, Ugal Kishore.

**Data curation**: Fnu Neha, Bismillah Athar Dar, Payal Bai, Zaghum Abbas, Abida Parveen.

**Formal analysis**: Fnu Neha, Bismillah Athar Dar, Payal Bai.

**Investigation**: Sadia Siddique, Sandeep Kumar Maheshwari, Fnu Neha, Kiran Lohana, Bismillah Athar Dar, Raja Pawan, Sitara Jabeen, Vikram Kumar, Abida Parveen.

**Methodology**: Muhammad Bari Hassan, Fnu Neelam, Fnu Neha, Bhawna Lohana, Kiran Lohana, Bismillah Athar Dar, Sitara Jabeen, Ali Karim, Zaghum Abbas.

**Project administration**: Fnu Neha, Kiran Lohana, Bismillah Athar Dar, Sitara Jabeen.

**Resources**: Fnu Neha, Kiran Lohana, Hina Kumari, Raja Pawan, Sitara Jabeen.

**Software**: Sadia Siddique, Bhawna Lohana, Hina Kumari, Sitara Jabeen, Ali Umair, Zaghum Abbas, Vikram Kumar.

**Supervision**: Fnu Neelam, Kiran Lohana, Hina Kumari, Ali Umair, Ali Karim, Ugal Kishore, Vikram Kumar.

**Validation**: Muhammad Bari Hassan, Fnu Neelam, Bhawna Lohana, Kiran Lohana, Hina Kumari, Raja Pawan, Payal Bai, Ali Umair, Ali Karim, Ugal Kishore.

**Visualization**: Fnu Neelam, Bhawna Lohana, Kiran Lohana, Bismillah Athar Dar, Hina Kumari, Raja Pawan, Payal Bai, Ali Umair, Ali Karim, Ugal Kishore, Vikram Kumar.

**Writing – original draft**: Muhammad Bari Hassan, Sadia Siddique, Sandeep Kumar Maheshwari, Fnu Neelam, Bhawna Lohana, Kiran Lohana, Bismillah Athar Dar, Hina Kumari, Raja Pawan, Payal Bai, Sitara Jabeen, Ali Umair, Ali Karim, Zaghum Abbas, Ugal Kishore, Vikram Kumar, Abida Parveen.

**Writing – review & editing**: Muhammad Bari Hassan, Sadia Siddique, Sandeep Kumar Maheshwari, Fnu Neelam, Bhawna Lohana, Kiran Lohana, Bismillah Athar Dar, Hina Kumari, Raja Pawan, Payal Bai, Sitara Jabeen, Ali Umair, Ali Karim, Zaghum Abbas, Ugal Kishore, Vikram Kumar, Abida Parveen.

## Supplementary Material
