# Supplementary material for: Chronic kidney disease and cardiovascular risk: Pathophysiology and interventional approaches – systematic review
Source: Medicine (Baltimore). 2026 May 12;104(49):e46189. doi: 10.1097/MD.0000000000046189 (PMC12689039; doi:10.1097/MD.0000000000046189)
Supplement: Supplementary file 1 [file medi-104-e46189-s001.docx]

Supplementary Table 1. Search Strategy for the Systematic Review

| **Database** | **Search Strategy** | **Date of Search** |
| --- | --- | --- |
| PubMed | ("Chronic Kidney Disease"[Mesh] OR "chronic kidney disease" OR "CKD") AND ("Cardiovascular Diseases"[Mesh] OR "cardiovascular risk" OR "coronary heart disease" OR "stroke") AND ("Pathophysiology" OR "Mechanism" OR "Intervention" OR "Management" OR "Therapy") | December 2024 |
| Embase | ('chronic kidney disease'/exp OR 'chronic kidney disease' OR 'CKD') AND ('cardiovascular disease'/exp OR 'cardiovascular risk' OR 'coronary heart disease' OR 'stroke') AND ('pathophysiology' OR 'mechanism' OR 'intervention' OR 'therapy') | December 2024 |
| Cochrane Library | ("chronic kidney disease" OR CKD) in Title Abstract Keyword AND ("cardiovascular disease" OR "cardiovascular risk" OR "coronary heart disease" OR "stroke") AND ("pathophysiology" OR "intervention" OR "therapy") | December 2024 |
| Additional sources | Manual search of references from included studies and relevant reviews | December 2024 |

**Supplementary Figure 1**: The **traffic light plot** for the risk of bias assessment, showing each study across the three Newcastle–Ottawa Scale domains (Selection, Comparability, Outcome/Exposure).


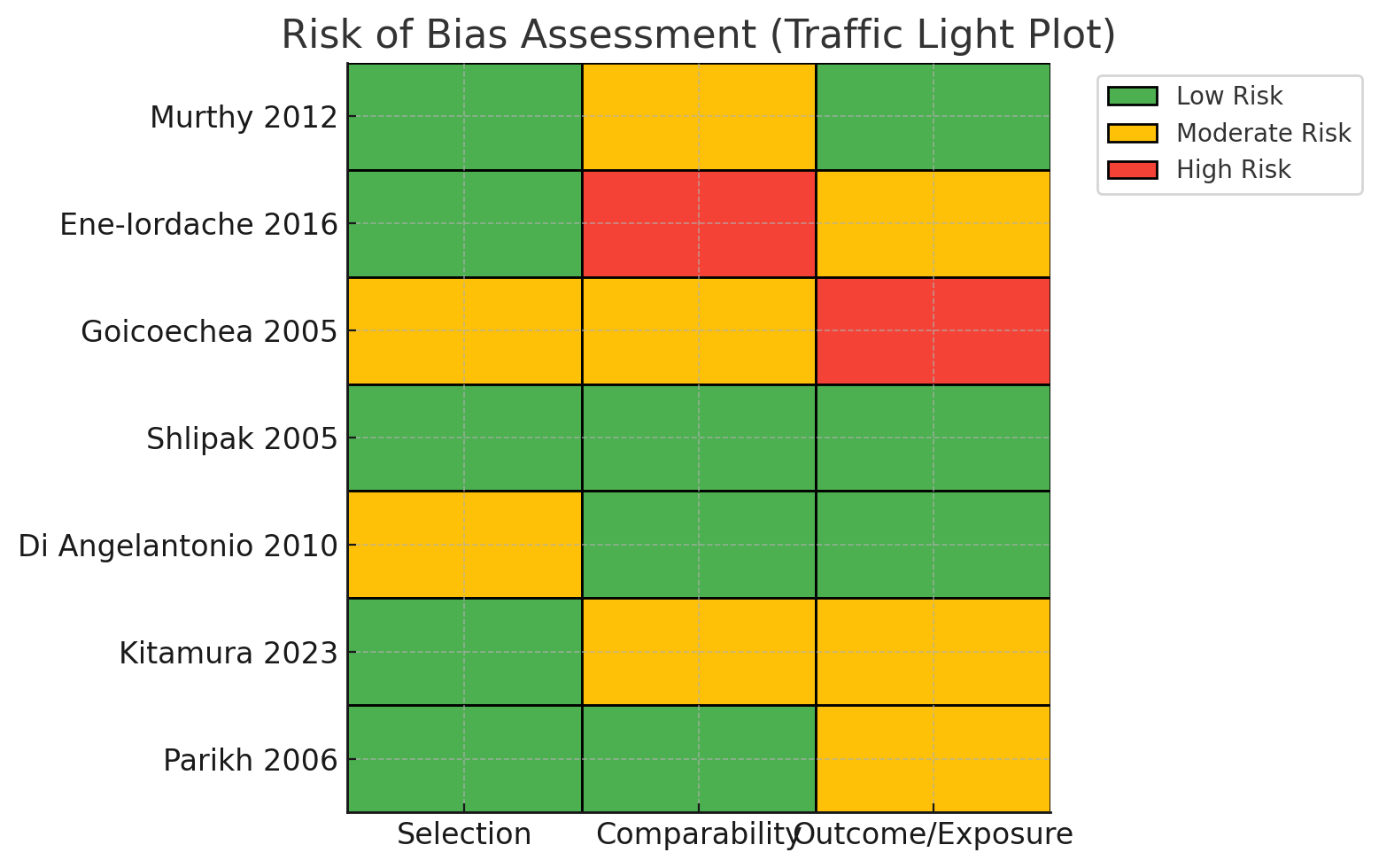


✅ Green = Low risk
🟨 Yellow = Moderate risk
🟥 Red = High risk
